# Can viable bacteria be present in the surface of ready-to-use surgical instruments?

**DOI:** 10.1590/0100-6991e-20253852-en

**Published:** 2025-11-24

**Authors:** CARLOS ROBERTO DE OLIVEIRA SAUER, FRANCISCO HIDEO AOKI, ROGÉRIO HAKIO KUBOYAMA, MÁRCIA REGINA SUZUKI DE LIMA, INÊS HELENA DE BARROS LEAL SARAIVA, CELY BARRETO SILVA, FRANCISCO AMÉRICO FERNANDES, MAURO JOSÉ DA COSTA SALLES

**Affiliations:** 1 - Secretaria Municipal de Saúde, Departamento de Saúde - Campinas - SP - Brasil; 2 - Faculdade de Ciências Médicas - Unicamp, Departamento de Clínica Médica - Campinas - SP - Brasil; 3 - Hospital Municipal Mário Gatti, Laboratório de Microbiologia Clínica - Campinas - SP - Brasil; 4 - Hospital Municipal Mário Gatti, Central de Material e Esterilização - Campinas - SP - Brasil; 5 - Hospital Municipal Mário Gatti, Serviço de Controle de Infecção Hospitalar - Campinas - SP - Brasil; 6 - Irmandade Santa Casa de Misericórdia de São Paulo - Hospital Central, Serviço de Controle de Infecção Hospitalar - São Paulo - SP - Brasi; 7 - Hospital Municipal Mário Gatti, Departamento de Cirurgia - Campinas - SP - Brasil; 8 - Escola Paulista de Medicina - UNIFESP, Laboratório Especial de Microbiologia Clínica (LEMC) - São Paulo - SP - Brasil

**Keywords:** Biofilms, Surgical Wound Infection, Surgical Instruments, Sterile Processing Department, Infection Control, Biofilmes, Infecção da Ferida Cirúrgica, Instrumentos Cirúrgicos, Centro de Material e Esterilização, Controle de Infecções

## Abstract

**Introduction:**

biofilm is considered a challenge regarding treatment of chronic diseases and, after a detailed observation of cleaning and sterilization processes, it is considered could be a threat to sterility of surgical instruments that are “ready to use”. Colored plastic bands (color coding tapes for marking surgical instruments) are frequently used to assist in the assembly of surgical instrument boxes. These bands form a lifting, which makes cleaning the material difficult. Epidemiological data regarding the frequency of surgical site infection in Brazil (up to 24% in Center-West Region) may be suggestive of contamination of operative instruments. The objective of this study is to answer the question: is there a risk of biofilm on ready-to-use surgical instruments?

**Methods:**

narrative literature review.

**Results:**

296 articles were found and a total of 163 were selected for detailed reading, of which 78 were included. During the survey, four knowledge domains were outlined: microbiology, pathophysiology/epidemiology, technology and management. This review pointed out the risk of the bacterial load prior to autoclaving, the efficiency of the sterilization method regarding the presence of microscopic soils and, under current conditions, the ability of the Material and Sterilization Centers to ensure adequate cleaning.

**Conclusion:**

after working extensively to associate all the collected information, there is a considerable probability of bacterial biofilms in ready-to-use surgical instruments and, therefore further research in this field of microbiology is justified, with an emphasis on improving process quality indicators, giving the potential impact on reduction of surgical site infection rates.

## RESUMO

**Introdução:**

biofilmes são considerados um desafio no tratamento de doenças crônicas e, após uma observação detalhada dos processos de limpeza e esterilização, podemos considerar que biofilmes podem representar uma ameaça à esterilidade dos instrumentos cirúrgicos prontos para o uso. É frequente o uso de fitas plásticas coloridas para auxiliar na montagem das caixas de instrumental cirúrgico, as quais formam um ressalto que dificulta a limpeza do material. Corroborando isso, temos dados epidemiológicos demonstram altas taxas de infecção de sítio cirúrgico, havendo regiões do Brasil cuja taxa chega a 24%. O objetivo deste estudo é responder a pergunta: é possível existir biofilme em instrumental cirúrgico pronto para uso?

**Método:**

revisão de literatura narrativa.

**Resultados:**

foram encontrados 296 artigos e selecionados, para leitura detalhada, um total de 163 artigos, dos quais foram incluídos 78 artigos. Durante o levantamento delineou-se quatro núcleos: microbiologia, fisiopatologia/epidemiologia, tecnologia e gestão. Artigos destas diferentes áreas de conhecimento indicam que a carga bacteriana prévia à autoclavagem, o comprometimento da eficiência do método de esterilização na presença de sujidades microscópicas e a dificuldade das Centrais de Material e Esterilização em assegurar a limpeza adequada deixam dúvidas sobre a garantia de esterilidade dos materiais cirúrgicos.

**Conclusão:**

há um considerável risco de existir biofilmes bacterianos em instrumental cirúrgico pronto para o uso, o que justifica maiores investimentos nessa área de pesquisa em microbiologia, com ênfase no aperfeiçoamento dos indicadores de qualidade do processo, tendo em vista o potencial impacto na redução das taxas de infecção do sítio cirúrgico.

## INTRODUCTION

Safe surgery depends on using sterile surgical instruments, which must meet the sterility assurance limit (SAL)^1^ ^,^ ^2^. Achieving this requires thorough cleaning^3^, but complete cleaning can be challenging due to features like folds, serrations, racks, lumens, and protrusions. The presence of protrusions is common in Brazil because colored plastic tapes are often used to identify surgical instruments; the tape color or sequence designates which instruments belong to a specific box, helping to reduce assembly time and streamline processes within the Material and Sterilization Center^4^ ^,^ ^5^.

Surgical instrument identification tapes are made of plastic, fixed by glue, and after numerous sterilization cycles in the autoclave, they deform or detach from the surgical instrument, which prevents cleaning and requires frequent inspection^5^. The requirement for inspections elevates the risk of microorganisms (such as bacteria and fungi) forming biofilm, as literature indicates challenges in maintaining an adequate number of trained personnel to consistently assure the quality of surgical instrument cleaning in Brazilian hospitals^6^. 

The presence of biofilm on dry surfaces of the Intensive Care Unit^7^ and on surgical instruments discarded at the end of their useful life has been described^8^. This is possible because biofilm is a bacterial or fungal life form with a great capacity for survival in adverse environments, displaying bacterial phenotypes that express high resistance to chemical agents^10^.

When considering the risk of biofilm in surgical material, the indicators of the process variables (temperature, time, and pressure) and the efficacy indicator (biological control) are of little use^3^ ^,^ ^11^, as there is doubt as to whether the high bacterial load of the instrument would allow SAL to be achieved and whether humid heat would be able to eradicate the biofilm^3^ ^,^ ^8^. Perhaps these facts explain what was observed with the implementation of the quality standards for surgical infection control of the National Institute of Health Care Excellence (NICE), which failed to reduce infection rates to less than 5%^12^. In Brazil, surgical site infection rate can reach up to 24% (Center-West region)^13^. Surgical site infections have a high humanitarian cost (perhaps higher than that of many wars) and are a major liability for governments^14^.

To answer the guiding question (“is there a risk of biofilm in ready-to-use surgical instruments?”), we conducted a literature review. At the beginning of the survey, in the search for keywords, we realized the need of a transdisciplinary approach, and during the reading of the texts we found that the answer required the in-depth investigation of four knowledge domains: microbiology, pathophysiology/epidemiology, technology, and management. 

## GOAL

The objective of this literature review is to evaluate the theoretical risk of the presence of viable bacteria in ready-to-use surgical instruments.

## METHODS

The transdisciplinary group, involved in this literature review, was composed of two pharmacists (clinical microbiologists), four infectious disease physicians, one surgeon, and one nurse (with extensive experience in Material and Sterilization Center). There was occasional collaboration with other nurses and a molecular biologist, however, most of the articles’ analysis was done jointly by a small team (pharmacist, nurse, and two infectious disease specialists). The main author (infectologist) was responsible for reviewing the borderline articles and distributing the activities (survey, reading abstracts, reading articles), and the inclusion of articles was done by consensus.

To exhaust the sources of information, we followed the steps:

1. Verification of keywords in the Virtual Health Library (DeCS - Health Science Descriptors);
2. Search in paid and free databases (PubMed, LILACS, SciELO, ScienceDirect, Google Scholar and Cochrane Central Register Trials), in English, Spanish or Portuguese, without time restriction;
3. Reverse search (use of the references of an article to reach other references);
4. Search for titles in the index of the SOBECC Journal (Brazilian Society of Nurses of the Operating Room, Anesthetic Recovery, and Material and Sterilization Center).

We thus used the aforementioned databases without restriction to the date of publication, using as keywords the following descriptors in health sciences (DeCS/BIREME): biofilms/biopelículas/biofilmes, infection control/control de infecciones/controle de infecções, cross infection/infección hospitalaria/infecção hospitalar, sterilization/esterilización/esterilização, surgical instruments/instrumentos quirúrgicos/instrumentos cirúrgicos, surgical site infection/infección de la herida quirúrgica/infecção de ferida operatória, equipment and supply labeling/etiquetado equipos y suministros/rotulagem de equipamentos e provisões, benchmarking, disinfection/desinfección/desinfecção.

Although there are no DeCS/BIREME descriptors for the concepts of “bacterial load” or “bioburden”, “processing of medical articles” and “material and sterilization center”, these terms were used in the research. Two Bireme descriptors (DeCS) showed null results in the search in the various bibliographic databases, benchmarking (“comparative evaluation in health care”) and disinfection/desinfección/desinfecção.

Inclusion criteria: data present that is relevant to the answer to the guiding question in at least one of the knowledge domains (microbiology, pathophysiology/epidemiology, technology, and management); most recent publication; unique contribution to the consolidation of some specific aspect necessary to answer the guiding question; contributes, in some way, to a transdisciplinary approach.

Exclusion criteria: unclear identification of the problem; lack of consistency between the guiding question and the chosen methodology; low relevance due to small sample size or limited coverage.

We read the abstracts of 296 articles located through the bibliographic survey. Through the application of the criteria, we selected 163 for full reading. After reading the full text, we excluded 85 articles, as we found that 78 met the inclusion criteria for this review.

Considering the extensive scope of the literature reviewed and recognizing the impracticality of employing a dedicated meta-analytic or integrative review , we have chosen to present the findings in a topical format.

## RESULTS

Throughout the process of reviewing the texts, it was possible to identify four knowledge domains that circumscribe the issue of biofilm risk in surgical instruments, namely: 1. Microbiology: studies on SAL and the subarea of bacterial biofilms; 2. Pathophysiology/Epidemiology: clinical aspects, distribution, and impact of surgical site infections; 3. Technology: description of the cleaning and sterilization processes of surgical material and prostheses; 4. Management: operation and current situation in Brazil of the Material and Sterilization Centers domains (MSCs). 

Of the 78 articles included in this study, observing the “number of knowledge centers” addressed by the publication, none of the articles addressed all the four knowledge domains described above, 12 addressed three centers, 37 addressed two, and 29 addressed a single knowledge center (Table 1).

**Table 1:** Distribution of articles according to the number of knowledge domains covered (1 to 4 centers).

Number of knowledge domains covered in the article	n	%
1	29	37.2
2	37	47.4
3	12	15.4
4	0	0
Total	78	100

The 64 articles published in English comprised 82% of the included articles, the 13 articles in Portuguese, 16.7%, and one article in Spanish corresponds to 1.3% (Table 2).

**Table 2:** Distribution of articles by language.

	n	%
English	64	82
Portuguese	13	16.7
Spanish	1	1.3
Total	78	100

When analyzing the nationality of the main author, we found that 14 scientific articles in English and 27 publications have a Brazilian as the main author (17,9% and 34,6% of the included references, respectively) (Table 3).

**Table 3:** Distribution of publications by Brazilian authors according to language compared to the total number of articles in the review (78 articles).

	n	%
English	14	17.9
Portuguese	13	16.7
Subtotal - Brazilian authors	27	34.6
Total	78	100

### 1. Microbiology

Bacteria have existed for 3.5 billion years on Earth and are the most abundant type of organism on the planet, with the sessile form (and not the planktonic form) as their main form of life^15^. This sessile form is biofilm, a consortium of extracellular matrix-producing microorganisms. It is composed of bacteria and fungi organized into strata and subpopulations, to allow specialization, like different tissues of multicellular organisms^16^. Capable of adhering to surfaces, they colonize medical devices (indwelling urinary catheters, venous catheters, orthopedic prostheses, endoscopes) and cause difficult-to-treat or recurrent infections (chronic otitis media, chronic osteomyelitis, periodontitis, infective endocarditis)^9^. Human diseases pathophysiology knowledge has evolved, as in the case of breast prosthesis contracture, which is attributed to the formation of biofilm, and anaplastic large cell lymphoma (ALCL), which is being correlated with the presence of the biofilm of the bacterium *Ralstonia sp*. in breast prostheses^17^. In the natural environment, biofilms play an important role at the base of the food chain of numerous habitats^18^. 

The formation of a biofilm begins with the conditioning of the surface through the deposition of proteins, which subsequently facilitates bacterial adhesion. This is followed by the maturation phase of the biofilm, when the three-dimensional structure is formed, with channels for hydration and successive layers. From this point forward, quorum sensing - a communication system among microorganisms within biofilms involving chemical or electrical stimuli - regulates the activities of the bacterial community. This process, among other functions, determines the timing of bacterial dispersion into the planktonic state, which constitutes the final phase of biofilm development^19^. 

The resistance to bactericidal agents arises from pathogens exhibiting low metabolic activity, specifically those classified as persistent or viable but non-culturable (VBNC) bacteria. The genes that determine these two phenotypes undergo stochastic suppression (random over time), allowing the bacteria to return to active metabolism and, thus, be susceptible to the action of antimicrobials^10^. This phenomenon clarifies how a cure is possible for diseases such as endocarditis through long courses of antibiotics. Another explanation for the high resistance of biofilms is the extracellular matrix itself, which prevents adequate levels of antimicrobial agents, protects bacteria from the body’s defense mechanisms, and favors the exchange of genetic information, even between different bacterial species. After seven days of growth, when the biofilm is mature, sessile bacteria can resist doses 500 to 5,000 times higher than bacteria of the same species in their planktonic phenotype^12^ ^,^ ^20^. 

The formation of biofilm on dry surfaces is a possible explanation for the high resistance to germicides and the survival for months of non-sporulated bacteria (in vegetative form) on hospital equipment and furniture^21^. Current knowledge of this novel biofilm conformation has advanced sufficiently to allow for the development of an experimental model for dry biofilm^22^. The accumulation of proteins, saline precipitates, and dirt favor bacterial adhesion in medical devices and protect microorganisms from the action of sterilants, including surgical instruments^23^ ^,^ ^24^.

### 2. Pathophysiology and Epidemiology

Surgical site infections (SSIs) are among the four most frequent healthcare-associated infections, along with pneumonia, urinary tract infections, and bloodstream infections^25^. Approximately 15% of all infections occurring in hospitalized patients are SSI^26^, and they may affect more than a third of surgical undergoing surgery in developing countries. When recently reviewing studies published between 1995 and 2015, the World Health Organization found an average rate of 11.2 surgical site infections per 100 patients operated on in developing countries^14^. In Brazil, the Department of Science and Technology of the Ministry of Health sponsored a study on the prevalence of healthcare-associated infections (HAIs) that was conducted from 2011 to 2013 and had the participation of 152 hospitals. In that study, surgical site infection corresponded to 1.5% of the total number of HAIs detected. If considering only patients who underwent surgical procedures, the prevalence was 9.8%. An important variation was observed when the data were stratified according to the Brazilian regions. The Center-West region had the highest SSI rate in patients undergoing surgery (24.2%) and the Southeast region had the lowest (8.2%). When the data analysis considered the characteristics of the institutions evaluated, it showed higher values in reference hospitals and in those with a number of beds equal to or greater than 200 (10.7% and 14.3%, respectively)^13^.

Importantly, surgical site infections are associated with prolonged length of hospital stay, the need for new surgical interventions to clean or debridement the lesions, in addition to causing a significant increase in the morbidity and lethality of surgical patients. In the USA, the costs associated with SSIs range from 3.5 to 10 billion dollars per year^26^. As a result, initiatives to prevent them have assumed particular importance for care institutions today.

For didactic purposes, surgical site infections are usually classified as superficial (when involving only skin or subcutaneous tissue below the incision) or deep (involving fascia and/or muscle tissues). When the infection reaches organs or spaces adjacent to the surgical site (for example, the meninges after surgery in the central nervous system), it is called organ-space infection. These infections usually appear within the first month after surgery, except when implants are placed (orthopedic prostheses, vascular grafts, prosthetic heart valves)^26^. In this case, an infection related to the surgical site is considered that occurring up to three months after implant surgery. 

SSI occurs more frequently in immunosuppressed patients or in those with important comorbidities, in particular diabetes mellitus. Factors related to the microorganisms involved (inoculum, virulence) as well as characteristics of the surgery performed (prolonged surgery time, presence of significant tissue damage, reoperation, presence of foreign bodies) also contribute to its appearance^25^. The intraoperative period is the moment of greatest risk for acquiring SSI, due to the exposure of the operative site to the patient’s skin flora or to microorganisms present in organs manipulated during surgery. It can also occur as a result of inoculation at the operative site of pathogens related to the focus of infection present at the time of surgery^25^. Therefore, it is important to carry out a careful anamnesis and a well-done physical examination in the preoperative period to rule out infectious conditions before elective surgery.

Finally, SSI may be secondary to exogenous sources of contamination, such as surgical material that is not adequately sterilized, dressings, or contaminated disinfectant solutions. There are outbreaks described in the medical literature relating surgical site infection to a team member colonized by a certain microorganism^27^ ^-^ ^31^.

### 3. Technology

The description of hygienic measures, such as hand hygiene, is very old. Charaka-Samhita, a Sanskrit medical textbook from the fourth century BC based on Vedic writings from the second millennium BC, prescribed “purity and cleanliness”^32^. 

In 2015, a 73% increase in the risk of patients acquiring nosocomial infection was found if their room was previously occupied by a patient colonized by some multidrug-resistant bacterium (enterococcus, *A. baumannii, S. aureus, or C. difficile*)^21^. In addition, the adequacy of the number of hygiene staff in a ward lead to a reduction in the infection rate by multidrug-resistant *S. aureus* by 30%^33^. Considering the impact of hygiene in the hospital environment, one can glimpse the importance of cleanliness in the control of surgical site infections, as the removal of dirt from surgical instruments is essential to ensure its sterility after autoclaving^34^ ^-^ ^36^.

The cleaning and sterilization process begins with pre-washing care, preventing the accumulation of large amounts of organic substances in the instruments and the drying out the material (keeping it moist during the procedure and/or immersing it as soon as possible in an aqueous solution of enzymatic detergent)^36^ ^,^ ^37^. 

This is followed by using the sonicator (ultrasound bath), manual cleaning, and putting the material through the thermo-disinfector. Once the material is cleaned, the instrument boxes are inspected, assembled, packaged with packaging for this specific use, and sterilized in an autoclave^38^ ^,^ ^39^. 

The presence of oil or dirt prevents the transfer of latent heat (a phenomenon in which water goes from a gaseous state to a liquid state, maintaining the temperature chosen for the operation of the autoclave, from 121°C to 134°C, which is responsible for the coagulation of bacterial intracellular proteins and consequent cell death. 

SAL is a theoretical concept that guarantees the probability of 1 spore in 1 million autoclaved instruments, if the physical parameters have been reached and the biological control is negative (i.e., all 10^6^ biological control spores have been killed during autoclaving)^1^ ^,^ ^2^. The spore is a bacterial form that is unable to multiply, but very resistant, capable of surviving humid heat (with a temperature of up to 100°C), ionizing radiation (at low doses), and ultraviolet radiation^43^. The experimental basis of SAL is the bacterial death curve observed when using a sterilant. 

For several decades, the bacterial load after the use of surgical instruments (pre-wash bacterial load) has been discussed, which is higher than 10^3^ bacteria per surgical instrument, with a flora represented by environmental bacteria and associated with the skin, in an amount that does not correlate with the potential for surgery contamination^44^ ^-^ ^49^. The washing of cannulated instruments was studied and a reduction in bacterial count was observed, although having evidenced recontamination by saprophytic and environmental bacteria^27^. After washing, the bacterial count varied among the various types of instruments, but ranged from 10^2^ to 10^3^ microorganisms^44^ ^,^ ^48^ ^,^ ^49^. The effect of the time elapsed between the end of surgery and material washing on the increase in bacterial load was studied, resulting in finding that the logarithmic bacterial growth phase begins in the sixth hour after the end of surgery^50^ ^,^ ^51^. 

Proteins, the main component of the dirt of surgical instruments^23^ ^,^ ^52^ ^-^ ^55^, are responsible for conditioning the surface of the instrument, the first step for bacterial adhesion to occur, which is the beginning of biofilm formation. Surgical materials with a complex design (Yankauer aspirator and cannulated instruments) were evaluated by direct inspection (fiberscope or partition of the instrument in half) and dirt was found in the material ready for use^24^ ^,^ ^56^ ^,^ ^57^. To document that debris in autoclaved instruments are not sterile, a study was designed in which pig bone plugs were inoculated with *Geobacillus stearothermophylus* spores and placed in cannulated materials. After autoclaving, the same species was recovered with the same pulsed-field electrophoresis (PFGE) pattern^56^.

### 4. Management

The Material and Sterilization Center (MSC) is considered a critical area of the hospital due to its importance in the control of healthcare-associated infections (HAIs)^58^ ^,^ ^59^ and is defined by the Brazilian Ministry of Health as a “functional unit located in health services intended for the processing of health products”^60^. 

In a survey to assess adherence to good practices in MSCs carried out by the National Health System in England, 29 units were evaluated, of which only 20% displayed “very good compliance” (greater than 80% adherence to good practices ) and 10% showed “good compliance” (between 61 and 80% adherence), that is, only 30% of the visited MSCs adhere to more than 60% of good practices^61^. In a study applying Lean Methodology, the MSC of Virginia Mason Medical Center (Seattle/WA) had the occurrence of three errors per 100 surgical procedures with risk of repercussions on infection rates^62^.

In Brazil, a relevant scientific production on MSC was stimulated with the creation, in 1991, of the SOBECC (Brazilian Society of Nurses of the Operating Room, Anesthetic Recovery, and Material and Sterilization Center), generating articles that compose a critical view of the Brazilian reality^63^. A 2016 study found that hospitals in the Center-West and North regions had the worst percentages of adherence to the standards of the National Health Surveillance Agency (ANVISA), around 50% (in the 75th percentile). In the other regions of Brazil, the percentage of adherence to good practices was around 80% (in the 75^th^ percentile)^64^. However, a master’s nursing dissertation that analyzed MSCs quality indicators in public hospitals accredited by the National Accreditation Organization (ONA) found that the MSCs evaluated presented “inconsistent” quality indicators and “there was no alignment of the actions with the strategic plan”^65^. Other articles report lack of investment in the MSCs, lack of ergonomics, reduced physical spaces, insufficient ventilation, poor visibility in the institutional sphere (by other sectors and the administration), untrained or insufficient staff, or inadequate staff selection process^6^ ^,^ ^66^ ^,^ ^67^.

An outbreak of infection with *Pseudomonas sp*. has been described associated with failure to sterilize surgical instruments during cataract surgery efforts carried out in 2016 in the state of São Paulo, with 22 infected people and one death^30^. Despite the reluctance to publish errors^29^, we found one report of an outbreak of infections by *Pseudomonas sp*. associated with arthroscopies and other infections by gram-positive cocci associated with orthopedic and ophthalmological procedures. All these events are associated with failures in the cleaning and sterilization process^31^ ^,^ ^68^. 

There are numerous publications addressing quality in the sterilization and cleaning process: one study evaluating the opportunities for hand hygiene in the MSC, other studies measuring the contamination of instruments with and without the use of gloves in the process of assembling boxes at MSC or the amount of surviving bacteria in challenges with spores in cannulated material, and some texts suggest the use of sterile water in the washing of instruments and even consider the microbiological techniques that determine sterility to be flawed^69^ ^-^ ^73^. 

In a publication found in the journal of the “Association of Operation Room Nurses” (AORN), the identification tape was considered a common practice in the USA. In this article, it is indicated that the tape, being porous, requires a prolonged sterilization time, presents a risk of fragmentation (possibility of generating a foreign body in the operative field) and, therefore, requires continuous monitoring of its condition^74^. 

Publications of adverse effects are unusual, even so, we found two reports of adverse effects associated with identification tapes: one associating their use with an outbreak of postoperative infection in vestibuloplasty and another with the description of a foreign body (fragment of tape) in an oroantral fistula repair surgery^75^ ^,^ ^76^. 

In 2016, a review on the subject was published suggesting that greater investment in research on the use of colored tapes to identify surgical materials is needed^77^. One article presented alternatives, such as the recording of QR code (“Quick Response”) or the use of radio frequency identification (RFID) system, which involve higher costs^78^.

## DISCUSSION

The multifaceted and broad result of this literature review, adequate to answer the question whether “it is possible to have biofilm in surgical instruments”, demanded an effort from the authors to conduct a truly transdisciplinary approach. 

We applied a methodology close to integrative review, and during the consolidation of the information it was possible to identify the four knowledge domains described above and fill the conceptual gaps of the articles of different knowledge centers. This resulted in greater accuracy and allowed a clear visualization of the risk of bacterial biofilms in ready-to-use surgical instruments. 

The need to include different areas of knowledge, both experimental work and epidemiological and conceptual studies, added to the originality of the proposal, prevented the use of informatics resources specific to the integrative review, and hindered the realization of a systematic review. In this situation, it is difficult to characterize any selection or publication bias due to the innovative aspect of the study, which is at the boundery of medical knowledge. Thus, in the epistemological roots of the present study, we find its strong points and its fragilities.

In general, articles from microbiology and disinfection/sterilization technology complement each other in demonstrating the resistance of biofilm in autoclave sterilization and allow the visualization of the theoretical risk of biofilm occurrence in surgical instruments. 

The pathophysiology/epidemiology articles demonstrate infection rates that could be explained by a “new” variable in the sequence of events that results in SSIs, and the management texts demonstrate that the available technologies/routines may not be being applied adequately, which magnifies the risk of the presence of biofilm in ready-to-use instruments. 

Finally, several articles indicate that the use of plastic tapes to identify the instruments and facilitate the assembly of the boxes makes cleaning difficult and can deform, increasing the risk of biofilm development. Perhaps, the routine of changing these tapes after shorter time intervals than those currently recommended can reduce this risk.

It was beyond the scope of this narrative review to establish an evident correlation between operative site infections and biofilm in surgical instruments. However, this study demonstrated the need to establish research protocols on the use of surgical instrument identification tapes and quality indicators of the cleaning/sterilization process of medical-hospital articles. 

At the confluence of these four knowledge domains, new evidence is emerging at an accelerated pace, so that it will soon be possible to use bibliographic review methodologies (integrative review and meta-analysis) with more specific objectives.

## CONCLUSION

The review of the pertinent literature showed that there is a risk of the presence of bacteria in ready-to-use surgical instruments.
